# Exploring Talent Cultivation of College Student-Athletes for New Ventures and Entrepreneurial Psychology of New Venture Entrepreneur

**DOI:** 10.3389/fpsyg.2021.679901

**Published:** 2021-08-12

**Authors:** Qinglei Wang, Mohd Salleh Aman, Lim Boon Hooi

**Affiliations:** Centre for Sport and Exercise Sciences, University of Malaya, Kuala Lumpur, Malaysia

**Keywords:** positive psychology, psychological capital, student athletes, career guidance curriculum for college students, promoting employment

## Abstract

To further promote the development of new ventures, sufficient research and analysis have been conducted on the managers (especially, new venture entrepreneurs) and employees of new ventures. In this study, the case investigation is adopted to study the psychological stress of new venture entrepreneurs, the psychological capital of college athletes and the cultivation of employability. The research results show that there is no significant difference in career guidance curriculum and employability among students with different academic performances (*p* > 0.05), but there is a significant difference in psychological capital among students with different academic performances (*p* < 0.05). The career guidance curriculum, employability, and psychological capital have different correlation degrees. The career guidance curriculum has a significantly positive impact on the employability of students, and psychological capital plays a mediating role in the impact of the career guidance curriculum on employability. The analysis of the psychological stress of new venture entrepreneurs indicates that the stress of the dimension of resource requirements is the least. Meanwhile, the psychological stress of new entrepreneurs has a positive impact on new venture performance. The research content is fully considered, which can provide a scientific and effective reference for the follow-up research.

## Introduction

Nowadays, innovation and entrepreneurship have become the center of social concern. The importance of nationwide entrepreneurship and innovation in national development has been realized by all (Nambisan et al., 2019). Every year, countless new ventures are emerging in China and are faced with serious survival challenges. Statistics imply that the average survival time of most new ventures is <3 years. Still, some successful new ventures have developed rapidly and become the main force in industrial and socio-economic development (Cunningham et al., 2019). Therefore, emphasis should be put on the analysis of the success and failure of new ventures. Enterprise development is greatly influenced by managers, as well as employees, so in-depth research on employees and managers should be conducted to explore the development path of new ventures (Ding, 2017). In terms of managers, especially, new venture entrepreneurs, they are faced with heavy challenges and psychological stress since new ventures are mostly in their infancy, with a short business establishment time and various uncertainties (Alvarez and Grazzi, 2018). With positive entrepreneurial stress coping strategies, entrepreneurs can reduce troubles, get in a better psychophysiological state, increase work efficiency, make better business decisions, and improve the performance of new ventures. However, too much entrepreneurial stress will harm entrepreneurial performance. Therefore, a correct and objective understanding of entrepreneurial stress is essential to enterprise development.

In terms of employees, college students are the main workforce in new ventures, and their employability is directly correlated with the development of new ventures. In particular, college athletes, being both athletes and students, are one special group among college students (Bosch et al., 2019). Compared with their peers, college athletes spend more time in daily training and competition, and their professional cultural knowledge is poor (Huml et al., 2017). Meanwhile, the employment situation of college athletes is more difficult than that of ordinary college graduates, so special attention should be paid to their employability (Wu et al., 2019). The cultivation of the employability of new venture entrepreneurs involves the cultivation of professional skills and abilities, as well as the psychological problems of college athletes. The employment psychology of college athletes is also affected by the popularization of higher education, resulting in many negative emotions, such as anxiety, depression, pessimism, imbalance, utilitarianism, and conformity. These negative emotions seriously affect the smooth employment of college athletes (Bamberger et al., 2018). The career guidance curriculum for college students based on positive psychology overcomes negative psychology with positive psychology and help college athletes establish the positive psychological quality, such as self-confidence, sense of achievement, optimism, calmness, rationality, and independent thinking (Kroshus et al., 2019). The employment quality education and psychological quality education of college athletes complement each other. The improvement of the employment quality increases the self-confidence, optimism, and other positive employment psychology of college athletes. Similarly, the improvement of psychological quality promotes the comprehensive employability of college athletes.

To promote the development of new ventures, the current situation of psychological factors of new venture entrepreneurs and college students is explored from the perspective of positive psychology in staff management and staff training. In this study, case analysis is adopted. Specifically, a questionnaire survey (QS) is designed for data collection, and the QS is issued to new venture entrepreneurs and college athletes. The influence of the career guidance curriculum is analyzed on the employment psychology of college athletes, and the relationship between the psychological status of new entrepreneurs and the performance of new ventures is explored. The purpose is to explore the psychological stress of different groups and to provide strategies for the development of new ventures from the perspective of psychology.

## Literature Review

### The Concept and Literature Review of Employability Training of College Athletes

Colleges and universities are a good choice for retired professional athletes to gain knowledge and skills for social employment, in which they can improve their comprehensive abilities through higher education and prepare themselves for a career (Corrada, 2020). Research has also found that the common problems of college athletes are insufficient knowledge reserves and limited employment options. Nearly 90% of student-athletes have similar career choices, indicating that the employment scope for student-athletes is small, with fewer employment choices (Bush, 2017). College athletes should have an optimistic attitude toward employment issues. When choosing a position, they should consider factors, such as salary level, nature of employment, location, and long-term development. Additionally, colleges and universities should provide more employment information and a career guidance curriculum for college athletes to help them find the ideal jobs (Knoester and Ridpath, 2020).

The cultivation of college athletes has always been the focus of research. Beauchemin (2014) analyzed the influence of college athletes on mental health and consultation awareness and attitude. Bird et al. (2018) studied the different psychological reactions of college athletes and non-student-athletes under stress. Kim et al. (2020) focused on analyzing the satisfaction and happiness of college athletes. In short, most of the existing research of college athletes only focuses on the psychological problems of college athletes, while research on the employment of college athletes is rare. Therefore, the research on the employability of college athletes is of great practical significance.

### Research Review on Entrepreneurial Stress and Venture Performance of New Venture Entrepreneurs

Entrepreneurial stress exists widely among entrepreneurs, which is brand-new stress different from work stress and arises from entrepreneurship. The cause of entrepreneurial stress is diverse including loneliness, indulging in work, interpersonal problems, and own psychological needs of entrepreneurs (Fadzil et al., 2019), as well as the huge risk factor. Heretofore, some researchers have analyzed the entrepreneurial stress of entrepreneurs. For example, Wiklund et al. (2020) discussed the importance of research on entrepreneurial mental health. Chienwattanasook and Jermsittiparsert (2019) discussed the impact of entrepreneurship education on entrepreneurial self-employment based on the survey data of Thai entrepreneurs.

New venture entrepreneurs are a special group of entrepreneurs, and the stress they face is undoubtedly huge. In short, there are few targeted studies on new venture entrepreneurs. Therefore, the psychological stress of new venture entrepreneurs should be focused on, especially, the relationship between the psychological factors of new venture entrepreneurs and new venture performance.

## Methods

### Analytical Methods

#### Career Guidance Curriculum Model for College Athletes

**Figure 1:**
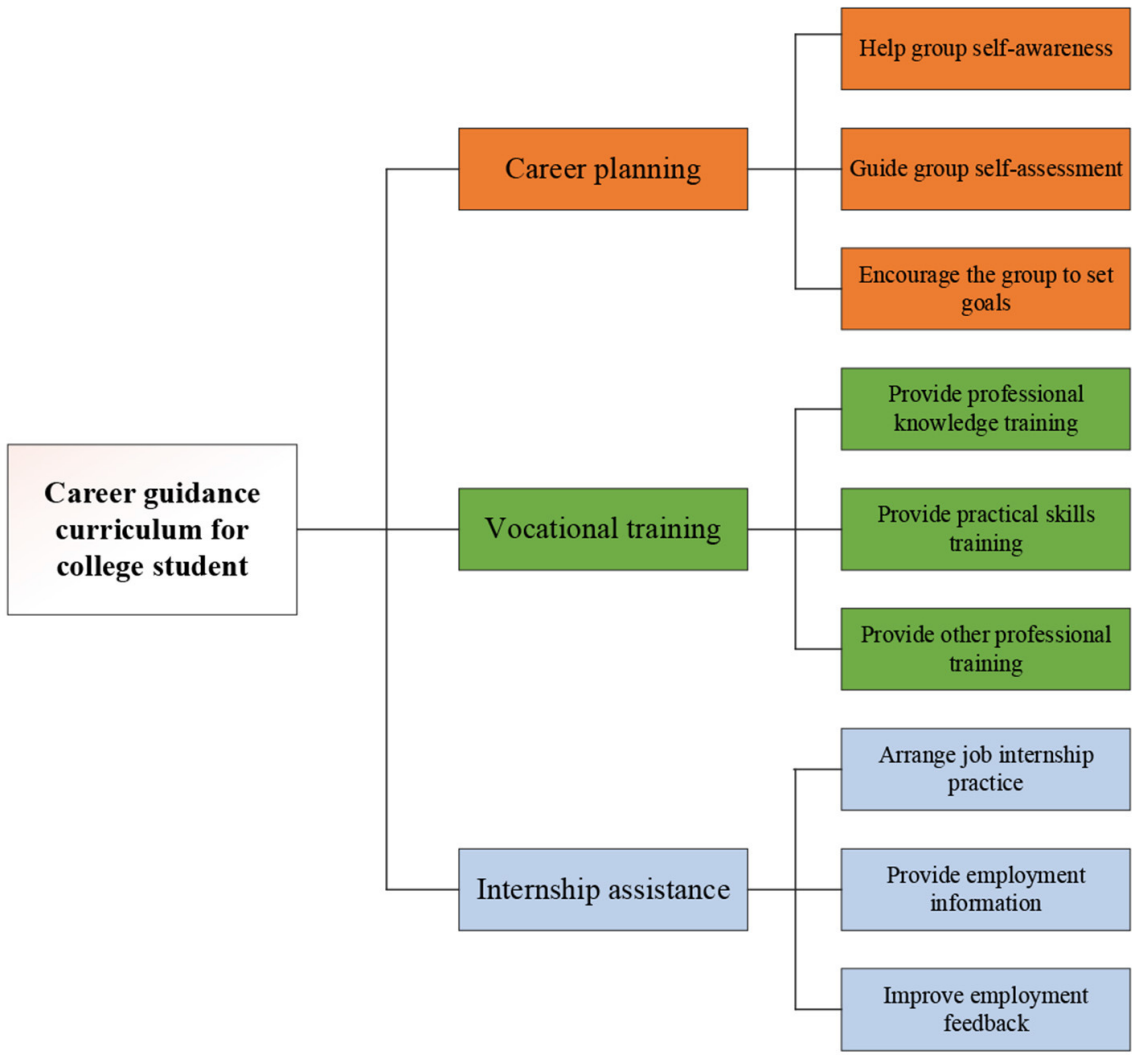
Structure of career guidance curriculum for college students.

The career guidance curriculum for college students is a powerful measure to promote the employment of graduates. In general, the career guidance curriculum should be offered for college athletes as early as their junior and senior years. Guided by these curricula, they can fully understand their career orientation, plan their every life and learning, and enhance their employment competitiveness (Deviants, 2017). The structure of the career guidance curriculum for college athletes is set into three dimensions based on the summary of the literature and previous research, combined with the actual situation. The detailed structural indicators are presented in Figure 1.

#### Design and Distribution of QS Scale for College Athletes

International and domestic researchers have not reached an agreement on the standard structure of psychological capital, and the commonly acknowledged are two-dimensional, three-dimensional, four-dimensional, and multi-dimensional structures. Chronologically, psychological capital has developed from two-dimensional and three-dimensional structures to four-dimensional and multi-dimensional structures. Based on different perspectives and backgrounds, the items of psychological capital QS vary. In this study, a psychological capital classification scale for college athletes is designed based on the Luthorne's psychological capital classification theory and actual situations. The main structure of the scale is as follows.

**Figure 2:**
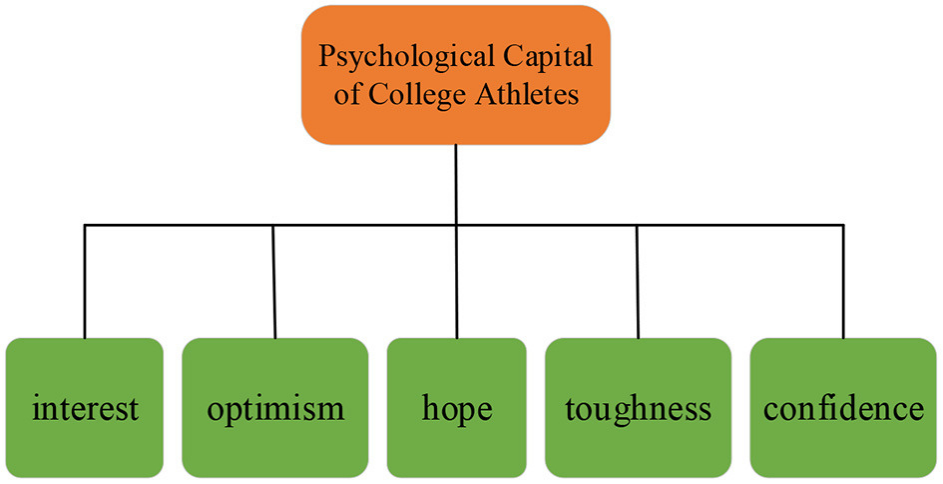
Measurement dimensions of psychological capital of college athletes.

Figure 2 shows that the psychological capital of college athletes encompasses five dimensions, namely, interest, optimism, hope, toughness, and confidence. Each dimension includes four items, totaling 20 items. The Likert 5-point scoring method is adopted in the scale. Specifically, five tiers of scores, 1, 2, 3, 4, and 5 points correspond to not matched, poorly matched, basically matched, matched, and well-matched, respectively.

A QS is designed and is conducted on the current situation of the psychological capital of college athletes in Zhejiang Province, China. The QS includes two parts, namely, pre-survey and formal survey. The designed QS is adapted from the positive psychological capital QS and used for college athletes. The QS contains 25 items and 5 dimensions, including confidence, toughness, hope, optimism, and interest. The Likert 5-point scale method is chosen.

A total of 250 QSs are issued, and 213 are recovered, of which 185 are valid QSs, with a response rate of 86.8%. The respondents are college athletes. Accordingly, the characteristics of their career guidance curriculum, psychological capital, and employability are measured. In this study, the designed QS is distributed and collected offline, and the athletes of the 2020 Zhejiang University Games are sampled for research. The students come from 12 colleges and universities across China, including Zhejiang University, Zhejiang Normal University, Ningbo University, and Hangzhou Normal University.

#### The Design and Distribution of the Psychological Stress Scale and Venture Performance Scale of New Venture Entrepreneurs

In this study, the psychological stress scale and venture performance scale of new venture entrepreneurs are divided into four parts, namely, the basic information scale of new venture entrepreneurs, the psychological stress scale of new venture entrepreneurs, the new venture performance scale, and the entrepreneurial stress management scale. The first part mainly includes eight indicators, namely, gender, age, education level, professional background, entrepreneurial role, establishment time, business type, and industrial nature. Based on the relevant research data and the actual situation, the psychological stress of new venture entrepreneurs (i.e., second part) is divided into four dimensions, namely, entrepreneurship involved, competitive intensity, resource requirements, and knowledge reserve. Each dimension has three items, with a total of 12 questions.

In the third part, the previous research results are cited to divide the new venture performance into three dimensions, namely, survival performance, growth performance, and development performance (Jin et al., 2017). Similarly, there are three items in each of the three dimensions of enterprise performance, with a total of nine questions. The enterprise survival performance measures whether the enterprise has been bankrupt and liquidated and the possibility of its continuous operation for over 8 years. Enterprise growth performance includes the growth of profitability, such as market share and number of employees, and the growth of financial indicators, such as sales and income. Enterprise development performance refers to the growth of the development potential of an enterprise, including product innovation ability, customer satisfaction, and brand recognition. Since it is difficult to obtain objective performance evaluation data, such as financial indicators, the subjective evaluation method is used for new venture performance measurement. The Likert 5-point scoring standard is also used in this QS. The higher the score is, the greater the psychological stress is.

The fourth part of the scale is the dimension of entrepreneurial stress management, which divides entrepreneurial stress into two dimensions, namely, active entrepreneurial stress management strategy and evasive entrepreneurial stress management strategy. Six items are designed for each dimension.

The QS is distributed both online and offline. The QS is focused on Hangzhou City, Zhejiang Province, China. Through online channels, new venture entrepreneurs in cities, such as Beijing, Xi'an, Nanjing, and Chengdu are investigated, a total of 500 QSs are distributed, and 458 are recovered, with a recovery rate of 91.6%.

#### Reliability and Validity of the Scale

The reliability analysis tests the consistency or reliability of the surveyed data. The reliability indicates whether each part of the scale measures a single concept and reflects the internal consistency of all items. Specifically, a Cronbach's alpha coefficient >0.8 indicates a high reliability of the data. If the coefficient is between 0.7 and 0.8, the reliability is good; if the coefficient is between 0.6 and 0.7, the reliability is acceptable. However, if the coefficient is <0.6, the reliability is poor. In terms of validity, the Kaiser–Meyer–Olkin (KMO) value and the Bartlett sphericity of the QS are examined. If the Bartlett sphericity test reaches a significant level and the KMO value is >0.8, the validity is high. If the KMO value is between 0.7 and 0.8, the validity is good; if the KMO value is between 0.6 and 0.7, the validity is acceptable. A KMO value <0.6 indicates a poor validity of the data. In this study, SPSS 25.0 software is used for the reliability and validity analysis (IBM, New York, USA).

### Theoretical Foundation

#### The Theoretical Concept of Employment Psychological Capital

In positive psychology, optimistic forces can overcome negative forces to make people happier. The theory of positive psychology provides new ideas for the employment of college athletes and career guidance curriculum of college students. Luthans put forward a new concept, psychological capital, by combining academic theories, such as human capital, organizational behavior, and positive psychology. After its proposal, psychological capital has been frequently applied in human resource management and personal career development (Witasari and Gustomo, 2020). Psychological capital is a positive psychological state, which is manifested throughout the growth and development of an individual. This positive psychological element is closely linked and applicable to the individual development of all age groups and can positively affect their long-term development. The psychological capital of college athletes can be improved through intervention on its particular dimensions to shift the way they think. As a result, the positive attitudes and behavioral effects of students can be brought into play, showing a theoretical significance for enriching the psychological capital theory, as well as a practical significance for improving the psychological status of individuals in their works and daily lives (Kirrane et al., 2017).

The academic community has proposed the theory of psychological capital intervention to improve the level of psychological capital of individuals or groups. Specifically, the mechanism of the psychological capital intervention is to intervene in issues, such as the enterprise inventory and quality of an individual. Meanwhile, the effectiveness of the model results has been confirmed in various studies worldwide, that is, psychological capital can positively affect individual efficiency and organizational performance (Ozturk and Karatepe, 2019). The career guidance curriculum is a bridge that helps students enter society smoothly by teaching them the social life and proficiencies needed in the practical works. In such a curriculum, internship, a practical link with the nature of workplace exercises, encourages students to contact employers and adapt to the new working environment, which can contribute to the cultivation of student employability. Job seekers can be provided with more career experiences and trial and error opportunities through multiple career guidance curriculums. Therefore, they can understand and identify the nature of an industry or a position, clarify and establish career goals, and enhance their recognition of the work values, thereby gaining a more positive attitude toward work acceptance. Thus, the satisfaction of student employability can be improved with the career guidance curriculum (Wang et al., 2018). Students can better examine and position their abilities through time investment; they can also reveal and test their psychological capital status. The efficient handling of various problems at work depends on a stable and powerful psychological capital, which can enhance the positive attitudes and beliefs in the work.

#### Hypothesis Model of Key Variables for Career Guidance Curriculum of College Students

Education of the employability view of college students includes three aspects, namely, the education of employment quality view, education of career selection and job search, and education of employment view. The employability quality education of college students includes employment quality education and employment psychological quality education (Manner, 2020). The employability quality education and psychological quality education of college athletes are complementary. The improvement of employability quality will increase the confidence, optimism, and other positive employment psychology of student athletes. Similarly, the improvement of psychological quality will improve the employability of college athletes. Therefore, the employment psychology of college athletes is also important to the research of career guidance curriculum for college students (Saito and Pham, 2020).

**Figure 3:**
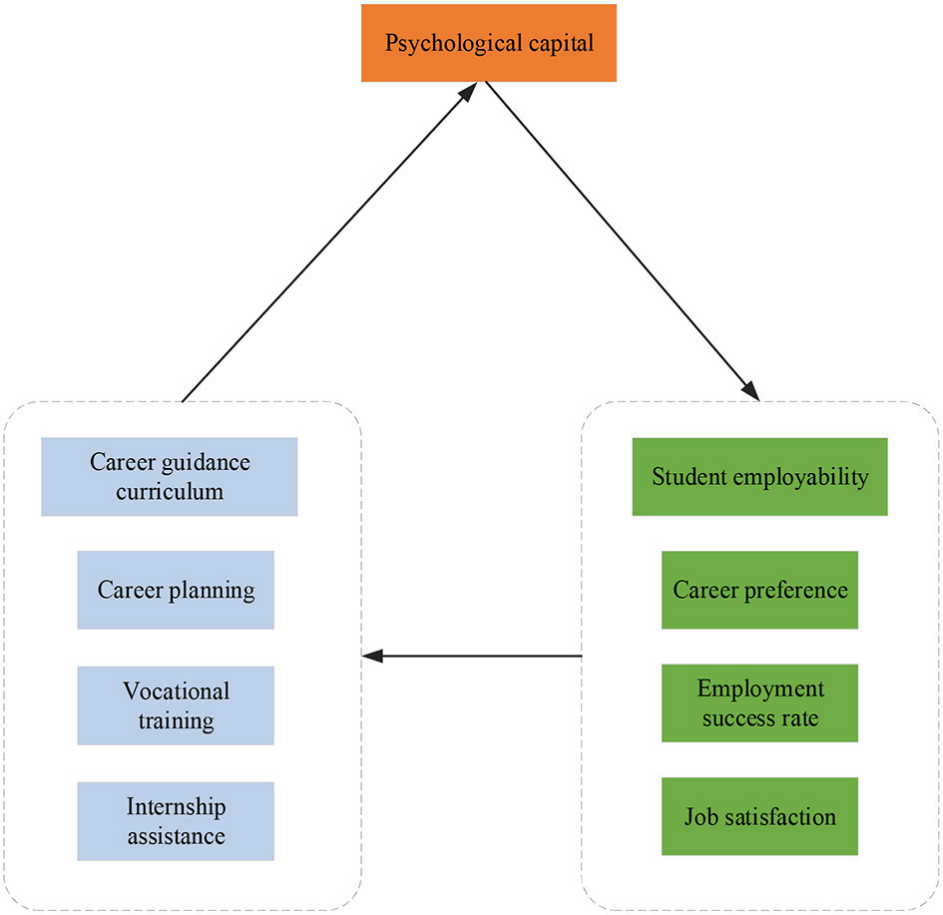
Key variable research model.

Ultimately, the employability and employment rates of college athletes can be improved through the career guidance curriculum. Based on the literature review, the effect of the career guidance curriculum for college students is researched empirically. The contextual characteristics of the mediating process of career guidance curriculum—psychological capital—of student employability will be explored to reveal the mechanism of career guidance curriculum on employment as comprehensively as possible, thereby enriching the theoretical and practical research of the influence of career guidance curriculum on employment. Figure 3 is the relationship hypothesis model of key variables.

Employment mentality and employment success rate of students are subjected to their ability reserves. Nevertheless, psychological capital can play a unique positive role in internship and employment, and students can cultivate and improve their psychological level through internship activities. College students can be provided with more career experiences and trial and error opportunities through multiple career guidance curricula. Therefore, they can understand and identify the nature of an industry or a position, clarify and establish career goals, and enhance their recognition of the work values, thereby gaining a more positive attitude toward work acceptance. While seeking a job, the psychological capital status of college students is based on their comprehensive perception and evaluation of the industry and position according to their earnings and contributions in the workplace. Sometimes, the confidence of students is damaged because they are not proficient in job skills, which affects their feelings and attitudes toward internships. At this time, the particular dimensions of student psychological capital can be intervened to adjust the corresponding attitudes and behaviors of the students. Hence, students can have a positive professional attitude and achieve efficient employment.

#### Research Hypothesis of Curriculum Guidance Education for College Students

Based on the above analysis, the following research hypotheses are proposed (Table 1).

**Table 1:** Research hypotheses.

**Hypotheses**	**Contents**
H1:	Career guidance curriculum for college athletes and college athletes' employability share a significantly positive correlation.
H1a:	Career planning of career guidance curriculum and employment success rate of student employability share a significantly positive correlation.
H1b:	Vocational training of career guidance curriculum and employment success rate of student employability share a significantly positive correlation.
H1c:	Internship assistance of career guidance curriculum and employment success rate of student employability share a significantly positive correlation.
H1d:	Career planning of career guidance curriculum and job satisfaction of student employability share a significantly positive correlation.
H1e:	Vocational training of career guidance curriculum and anticipated job satisfaction of student employability share a significantly positive correlation.
H1f:	Internship assistance of career guidance curriculum and job satisfaction of student employability share a significantly positive correlation.
H1g:	Career planning of career guidance curriculum and career preference of student employability share a significantly positive correlation.
H1h:	Vocational training of career guidance curriculum and career preference of student employability share a significantly positive correlation.
H1i:	Internship assistance of career guidance curriculum and career preference of student employability share a significantly positive correlation.

#### The Model of the Psychological Stress of New Venture Entrepreneurs and New Venture Performance

**Figure 4:**
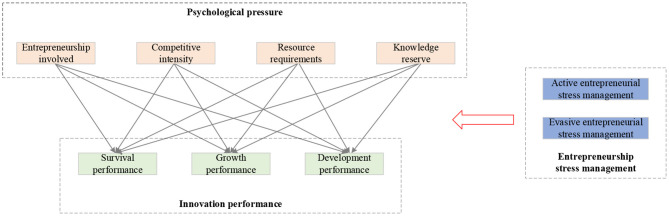
The model of psychological stress and new venture performance of new venture entrepreneurs.

Based on the above analysis, the entrepreneurial stress of new venture entrepreneurs is set as the independent variable, and the new venture performance is taken as the dependent variable. Meanwhile, the entrepreneurial stress management strategy is introduced as the moderating variable to explore the correlation between the entrepreneurial stress of college athletes and its four dimensions, and the new venture performance, as well as the moderating role of entrepreneurial stress management strategy in the correlation of each dimension. The specific results of the research model are shown in Figure 4.

## Results

### The Statistics of the Basic Situation of the New Venture Entrepreneurs, Psychological Stress Model, and New Venture Performance

**Figure 5:**
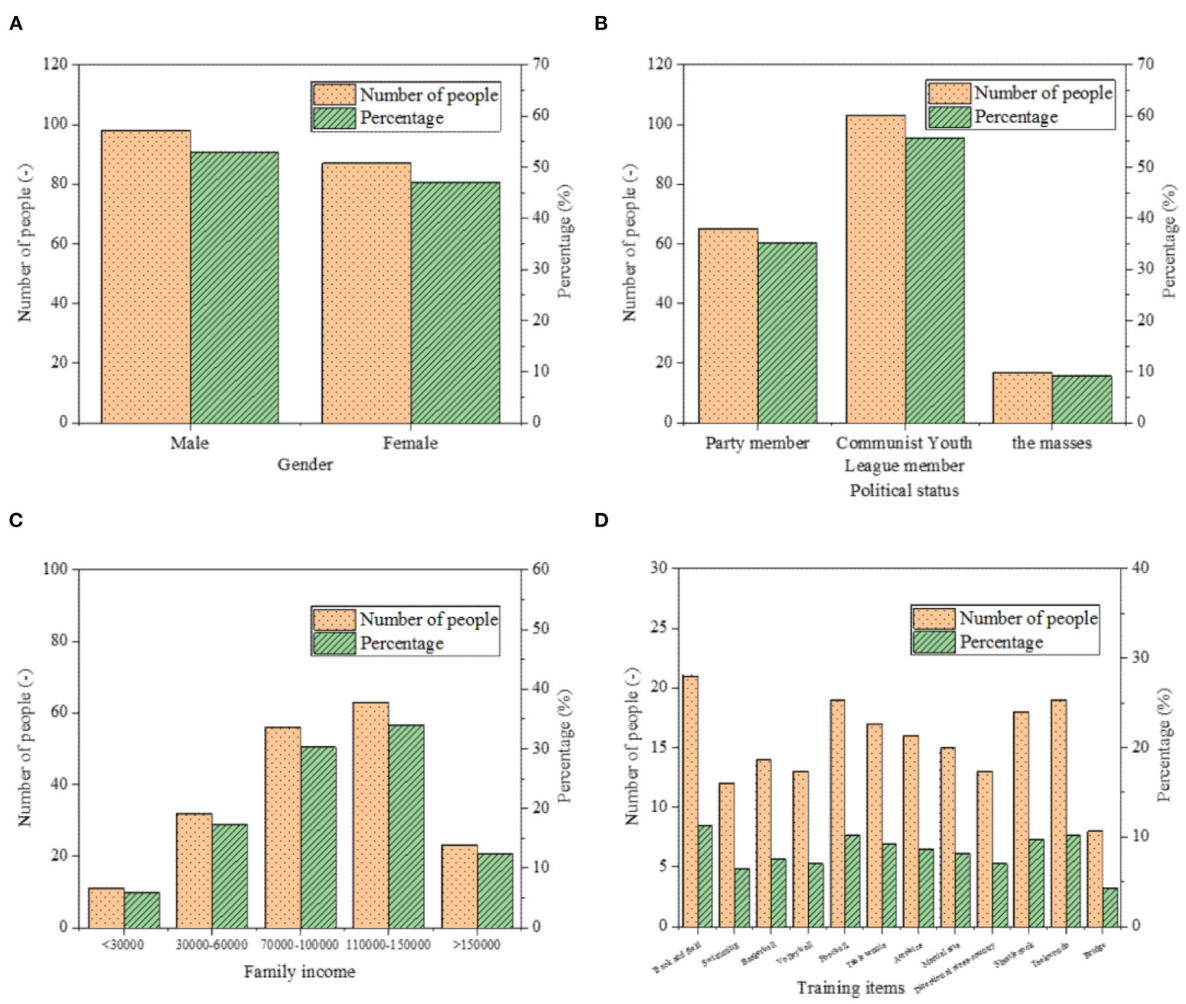
Statistics of the basic situation of the investigated college athletes: **(A)** gender; **(B)** political status; **(C)** family income, and **(D)** training program.

First, the situation of the surveyed college athletes is summarized from the aspects of gender, political status, family income, and training items. The specific situation is shown in Figures 5A–D.

Among all the subjects, male and female accounts for 52.97 and 47.02%, respectively. Sports events cover 12 items, such as track and field, swimming, basketball, volleyball, football, table tennis, aerobics, martial arts, directional cross-country, shuttlecock, taekwondo, and bridge. Of these items, the number of people in the track and field project is the largest, which is 21, accounting for 11.35%; the number of people in the Bridge Project is the smallest, which is 8, accounting for 4.32%.

**Figure 6:**
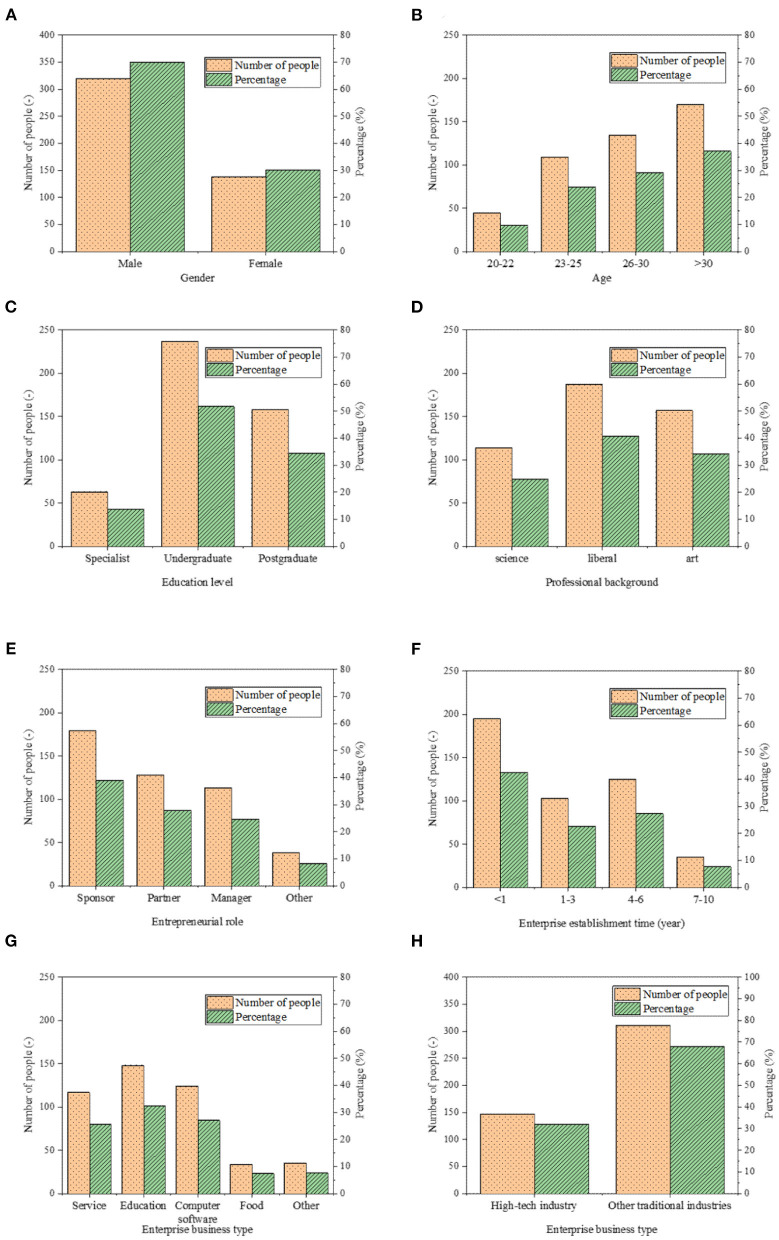
Statistics of the basic information of new venture entrepreneurs and new ventures: **(A)** gender; **(B)** age; **(C)** education level; **(D)** professional background; **(E)** entrepreneurial role; **(F)** enterprise establishment time; **(G)** enterprise business type; and **(H)** enterprise industrial nature.

The basic situation of new venture entrepreneurs is shown in Figure 6. Figures 6A–H correspond to the gender, age, education level, professional background, entrepreneurial role, enterprise establishment time, enterprise business type, and enterprise industrial nature, respectively.

Figure 6 suggests that male entrepreneurs account for 69.87% of the total respondents. Most new venture entrepreneurs are over 30 years old. The education level of new venture entrepreneurs is generally high, with 86.24% of them having a bachelor's degree or above. In terms of professional background, there are more entrepreneurs with liberal arts and art backgrounds. Most new venture entrepreneurs start their businesses as founders. Meanwhile, 42.58% of the investigated new ventures have been established for less than a year. The business types of new ventures are extensive, and there are many new ventures in the education industry. Additionally, 32.09% of the surveyed new ventures belong to high-tech industries.

### Reliability and Validity of the Scale

(1) Analysis on the QS scale of the college athletes

The reliability and validity of the career guidance curriculum scale, psychological capital scale, student employability scale, psychological capital scale, and the total QS are analyzed, and the results are summarized in Table 2.

**Table 2:** Reliability analysis results of the questionnaire survey (QS).

**Dimensions**	**Cronbach's alpha**	**Number of items**
Career guidance curriculum	0.852	10
Psychological capital	0.847	10
Student employability	0.902	10
Total QS	0.845	35

Table 2 illustrates that the Cronbach's alpha coefficients of career guidance curriculum scale, psychological capital scale, the student employability scale, and the total QS are 0.852, 0.847, 0.902, and 0.845, respectively. Thus, the Cronbach's alpha coefficients of all the QS scales are all >0.8, indicating that the data have excellent reliability and can be submitted to subsequent analysis.

The validity of the QS is shown in Table 3.

**Table 3:** Validity analysis results of the QS.

**Dimensions**	**KMO**	**Approximate chi-square**	**Sig**.
Career guidance curriculum	0.812	254.536	0.000
Psychological capital	0.883	145.943	0.000
Student employability	0.861	859.638	0.000

The KMO values of student employability scale, psychological capital scale, and career guidance curriculum scale for college athletes are all >0.8. Moreover, the results of the Bartlett sphericity test have reached significant levels, indicating that the validity is acceptable, and the variables are structured and correlated. Hence, the factor analysis can be performed.

(2) Reliability and validity of the psychological stress scale and new venture performance scale of new entrepreneurs.

In this study, the reliability and validity are verified for each dimension of the psychological stress scale and entrepreneurial performance scale of new venture entrepreneurs. The pairing results of the reliability analysis are shown in Table 4.

**Table 4:** Reliability analysis of the scale.

**Dimensions**		**Cronbach's alpha coefficient**	
Psychological stress	Entrepreneurship involved	0.847	0.821
	Competitive intensity	0.824	
	Resource requirements	0.803	
	Knowledge reserve	0.792	
New venture performance	Survival performance	0.863	0.825
	Growth performance	0.782	
	Development performance	0.811	
Entrepreneurial stress management	Active entrepreneurial stress management	0.817	0.811
	Evasive entrepreneurial stress management	0.801	

Table 4 shows that the Cronbach's alpha coefficients of each dimension in the new venture entrepreneur psychological stress scale, entrepreneurial performance scale, and psychological stress management scale are >0.7, which indicates that the scale has passed the reliability test and can be used for subsequent research and analysis.

The results of the validity test are shown in Table 5.

**Table 5:** Validity analysis results of the QS.

**Dimensions**	**KMO**	**Approximate chi-square**	**Sig**.
Psychological stress	0.834	268.25	0.000
New venture performance	0.817	179.57	0.000
Entrepreneurial stress management	0.808	547.25	0.000

Table 5 indicates that the KMO values of each scale are >0.8, indicating that the correlation between the variables is good, which can be applied to the follow-up study.

### Analysis of the Related Factors of Employability of College Athletes

The single-factor ANOVA is employed to examine whether there are differences in career guidance curriculum, employability, and psychological capital of students of different genders, different political status, and different family incomes (Table 6).

**Table 6:** Single-factor ANOVA of each variable.

**Items**	**Variables**	***F*-values**	***p*-values**
Career guidance curriculum	Gender	1.453	0.431
	Political status	1.667	0.732
	Family income	2.142	0.817
Psychological capital	Gender	0.855	0.552
	Political status	0.649	0.435
	Family income	0.792	0.604
Student employability	Gender	0.496	0.732
	Political status	0.598	0.304
	Family income	0.302	0.295

Table 6 indicates that the *p*-values of all the statistical items are >0.05, indicating that all items have not passed the significance test, so the gender (male and female), the political status (the member of the Communist Youth League of China, the member of the Communist Party of China, and the mass), and family income of college athletes have no statistical differences in the career guidance curriculum, psychological capital, and student employability perspectives (*p* > 0.05).

The single-factor ANOVA is practiced again to test whether there are differences in career guidance curriculum, employability, and psychological capital for students with different academic performances (Table 7).

**Table 7:** Variable analysis of different academic performances.

**Items**	**Academic performance**	**Mean value**	**Standard deviation**	***F*-values**	***p*-values**
Career guidance curriculum	Top 20%	2.98	0.763	1.638	0.203
	Top 20–60%	3.17	0.821		
	Top 60–100%	2.81	0.683		
Psychological capital	Top 20%	3.56	0.692	3.493	0.028
	Top 20–60%	3.25	0.756		
	Top 60–100%	2.97	0.843		
Student employability	Top 20%	3.12	0.681	2.786	0.082
	Top 20–60%	3.26	0.733		
	Top 60–100%	2.83	0.825		

According to Table 7, students with different academic performances have no statistical differences in career guidance curriculum and employability (*p* > 0.05). Moreover, students with different academic performances have statistical differences in psychological capital (*p* < 0.05), and those whose academic performances are at the top 20% have the highest psychological capital scores. College athletes with excellent academic performance have higher scores of psychological capital, confidence, hope, toughness, and optimism. Overall psychological capital and confidence of college students with excellent academic performance are significantly different from those who get poor academic performance. Specifically, the more confident a student is, the higher his/her academic performance is, so psychological capital can predict the academic performance of college students.

### Analysis of Key Variables of College Athletes

The Pearson's correlation analysis is employed to analyze the relationships among student employability, psychological capital, and career guidance curriculum. According to the directions, when the correlation coefficient between variables is >0.5, the correlation is considered to be significant; when the correlation coefficient between variables is >0.8, the correlation is considered to be highly significant. The detailed results are summarized in Table 8.

**Table 8:** Correlation analysis of key variables.

**Dimensions**	**Career guidance curriculum**	**Psychological capital**	**Student employability**
Career guidance curriculum	1		
Psychological capital	0.578^**^	1	
Student employability	0.639^**^	0.743^**^	1

** *Indicates correlation at the 0.01 significance level*.

The analysis results show that the correlation coefficient between career guidance curriculum for college athletes and psychological capital is 0.578, which is significant at the level of 0.000, indicating that career guidance curriculum is positively correlated with psychological capital. The correlation coefficient between career guidance curriculum and student employability is 0.639, which is significant at the level of 0.000, indicating that career guidance curriculum is positively correlated with student employability. The correlation coefficient between psychological capital and student employability is 0.743, which is significant at the level of 0.000, indicating that psychological capital is positively correlated with student employability.

Based on the significantly positive correlation between the career guidance curriculum and student employability, the Pearson's correlation analysis is practiced again to explore the correlations between the various factors of career guidance curriculum and student employability. The analysis results are summarized in Table 9.

**Table 9:** Correlation analysis of factors of key variables.

**Terms**	**Career planning**	**Vocational training**	**Internship assistance**	**Career preference**	**Employment success rate**	**Job satisfaction**
Career planning	1					
Vocational training	—	1				
Internship assistance	—	—	1			
Career preference	0.437^**^	0.314^**^	0.459^**^	1		
Employment success rate	0.483^**^	0.389^**^	0.596^**^	—	1	
Job satisfaction	0.426^**^	0.451^**^	0.527^**^	—	—	1

** *Indicates correlation at the 0.01 significance level*.

The analysis results show that the correlation coefficients between the three factors of career guidance curriculum (i.e., career planning, vocational training, and internship assistance) and career preference are 0.437, 0.314, and 0.459, respectively, which are significant at the level of 0.000, indicating that career planning, vocational training, and international assistance are positively correlated with career preference. The correlation coefficients between the three factors of career guidance curriculum (i.e., career planning, vocational training, and internship assistance) and employment success rate are 0.483, 0.389, and 0.596, respectively, which are significant at the level of 0.000, indicating that career planning, vocational training, and internship assistance are positively correlated with the employment success rate. The correlation coefficients between the three factors of career guidance curriculum (i.e., career planning, vocational training, and internship assistance) and the anticipated job satisfaction are 0.426, 0.451, and 0.527, respectively, which are significant at the level of 0.000, indicating that career planning, vocational training, and internship assistance are positively correlated with the anticipated job satisfaction.

### Analysis of the Relationship of Psychological Stress, Stress Management, and New Venture Performance of New Venture Entrepreneurs

**Figure 7:**
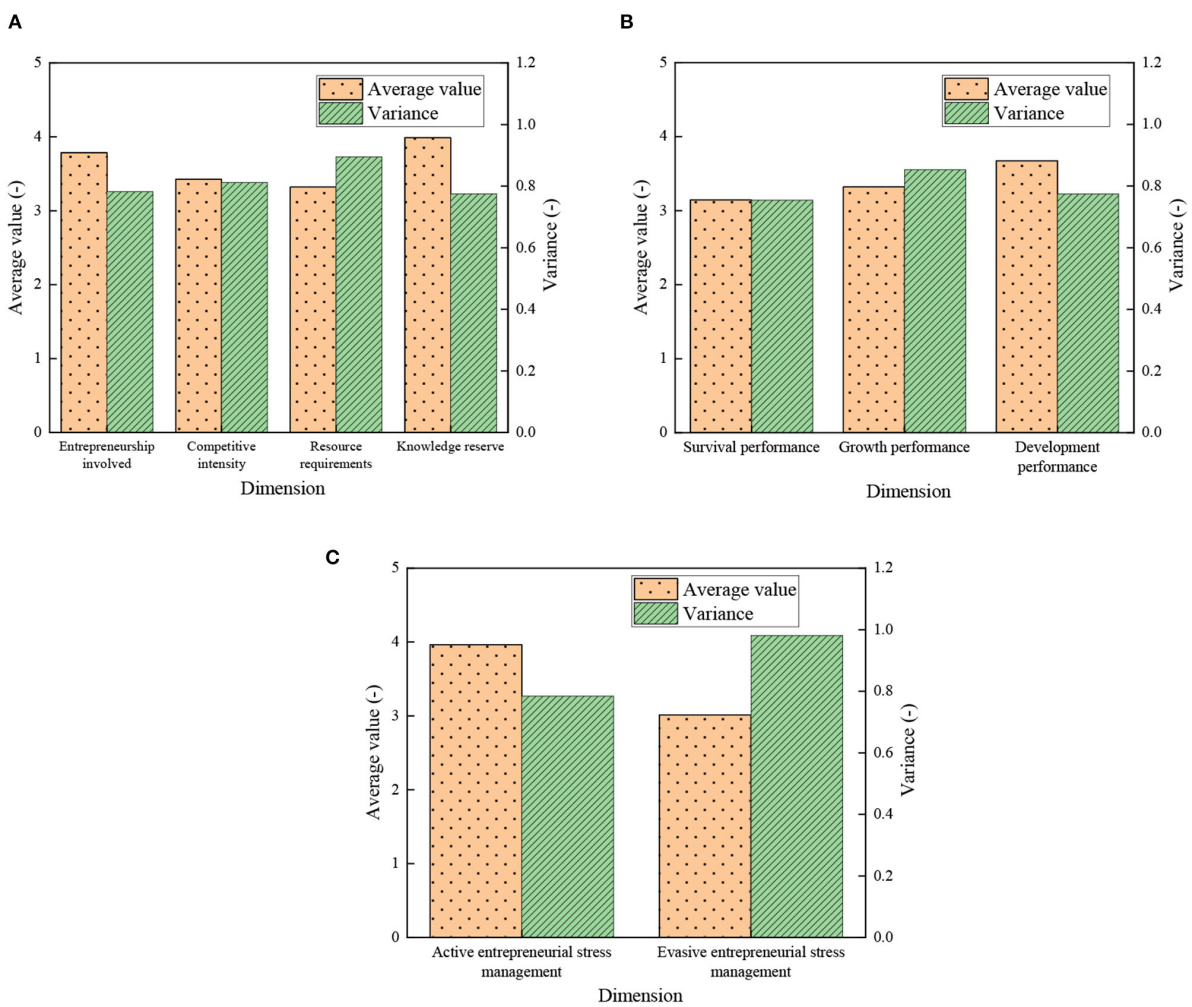
Data statistics of psychological stress, stress management, and new venture performance of new venture entrepreneurs: **(A)** psychological stress statistics of new venture entrepreneurs; **(B)** the statistics of enterprise performance; and **(C)** the score statistics of psychological stress management scale.

In this study, the specific QS survey results of the psychological stress, stress management, and venture performance of new venture entrepreneurs are statistically analyzed and shown in Figure 7: Figure 7A displays the statistical score of the psychological stress scale of new venture entrepreneurs, Figure 7B shows the score of the new venture performance scale, and Figure 7C is the score statistics of psychological stress management scale.

Figure 7A indicates that of the four dimensions of psychological stress of new venture entrepreneur, the resource requirements get the lowest score (3.321) and the largest variance (0.895); the knowledge reserve dimension gets the highest score (3.988) and the lowest variance (0.782); the average total score is 3.630. Figure 7B implies that of the three dimensions of new venture performance, the score of development performance is significantly higher than the other two dimensions, with a score of 3.674; the survival performance gets the lowest score (3.145). The growth performance gets the largest variance (0.853), and the survival performance gets the smallest variance (0.754). Figure 7C suggests that of the two dimensions of entrepreneurial stress management, the score of active entrepreneurial stress management is 3.965, which is significantly higher than that of evasive entrepreneurial stress management. The variance of the evasive entrepreneurial stress management dimension is 0.981, which is higher than that of the active entrepreneurial stress management dimension.

Subsequently, the relationship between entrepreneurial stress and new venture performance is analyzed, and the specific results are shown in Table 10.

**Table 10:** The correlation analysis of entrepreneurial stress and new venture performance of new venture entrepreneurs.

	**1**	**2**	**3**	**4**	**5**	**6**	**7**
1 Entrepreneurship involved	1	0.147	0.114	0.188	0.358^**^	0.367^**^	0.314^**^
2 Competitive intensity		1	0.147	0.147	0.314^**^	0.387^**^	0.308^**^
3 Resource requirements			1	0.159	0.325^**^	0.357^**^	0.325^**^
4 Knowledge reserve				1	0.357^**^	0.342^**^	0.347*8
5 Survival performance					1	0.201	0.198
6 Growth performance						1	0.214
7 Development performance							1

** *Indicates significance at 0.01 level, N = 485*.

Table 10 shows that entrepreneurship involved, competitive intensity, resource requirements, and knowledge reserve are positively correlated with enterprise survival performance, enterprise growth performance, and enterprise development performance at the significant level of 0.01. Thus, entrepreneurship involved, competitive intensity, resource requirements, and knowledge reserve can positively affect new venture performance. Hence, the greater the stress of entrepreneurship involved, competitive intensity, resource demand, and knowledge reserve that are perceived by new venture entrepreneurs, the higher the new venture performance is.

Meanwhile, the regression analysis is performed on each dimension of entrepreneurial stress and new venture performance of new venture entrepreneurs, and the specific results are shown in Table 11.

**Table 11:** Regression analysis of entrepreneurial stress dimensions and new venture performance of new venture entrepreneurs.

**Model**		**Non-standardized coefficient**		**Standard error**	***t***	**sig**	***R*^**2**^**	***F***
		**B**	**Standard error**	**Standard coefficient**				
Survival performance	Entrepreneurship involved	0.257	0.059	0.249	4.825	0.000	0.217	23.471
	Competitive intensity	0.142	0.057	0.124	0.984	0.007		
	Resource requirements	0.187	0.055	0.175	2.987	0.007		
	Knowledge reserve	0.298	0.054	0.274	4.574	0.000		
Growth performance	Entrepreneurship involved	0.185	0.048	0.198	3.714	0.000	0.257	29.547
	Competitive intensity	0.194	0.047	0.201	3.869	0.000		
	Resource requirements	0.185	0.045	0.198	3.997	0.000		
	Knowledge reserve	0.152	0.047	0.147	2.824	0.000		
Development performance	Entrepreneurship involved	0.247	0.054	0.218	4.147	0.000	0.258	28.214
	Competitive intensity	0.286	0.051	0.299	5.654	0.000		
	Resource requirements	0.047	0.052	0.087	0.854	0.000		
	Knowledge reserve	0.198	0.050	0.198	3.147	0.007		

Table 11 implies that the regression coefficients of entrepreneurship involved, competition intensity, resource requirements, and knowledge reserve of new venture entrepreneurs with the survival performance, growth performance, and development performance of new ventures are 0.249, 0.124, 0.175, 0.274, 0.198, 0.201, 0.198, 0.147, 0.218, 0.299, 0.087, and 0.198, respectively, with significant values <0.05. The adjusted *R*^2^ of the judgment coefficient is 0.217/0.257 and 0.258, respectively. The results show that the entrepreneurship involved, competition intensity, resource requirements, and knowledge reserve have positive effects on the survival performance, growth performance, and development performance of new ventures.

## Discussion

The analysis of the QS results indicates that new venture entrepreneurs show the following characteristics: average age distribution, high education level, and rich professional background. Meanwhile, most of the new venture entrepreneurs start their businesses as founders, which further reflects the activity of nationwide entrepreneurship. Besides, at the present stage, many new ventures are high-tech industries, and the development of new science and technology ventures promotes market prosperity and economic development.

Yet, the age of college athletes is relatively low, so it is necessary to cultivate their comprehensive employability. The test suggests that the correlation coefficient between career guidance curriculum and student employability is 0.639, showing a significant level of 0.000, which reveals a significantly positive correlation between career guidance curriculum and student employability. Thus, hypothesis H1 is valid. The correlation analysis of key factors of college career guidance curriculum and student employability indicates that key factors are significantly correlated at the 0.01 level. Through the correlation analysis of the key variables, the analysis results show that there is a significant correlation between the factors at the 0.01 level. The career planning, vocational training, and internship assistance of the career guidance curriculum are positively correlated with the employment success rate, career preference, and job satisfaction of student employability. Hence, the research hypotheses are valid. The influence of psychological factors of college athletes on sports level and training effect is generally concerned in the past, while the research on the employment of college athletes is rare [27–28]. In comparison, in this study, the influence of psychological factors of college students on their employment cognition is analyzed, adhering to the comprehensive development of college athletes, which has very innovative and practical significance.

The entrepreneurial stress of new venture entrepreneurs can positively affect the new venture performance, so their entrepreneurial stress should be optimized through positive entrepreneurial stress management measures, thereby improving the new venture performance. While new ventures should change the negative treatment toward entrepreneurship involved, they should exert strong innovation ability to produce more outstanding products in the fierce competition, and entrepreneurs and ventures as a whole should renovate ideas and treat the stress of knowledge reserve as an opportunity for knowledge renewal and ability enhancement.

## Conclusion

In this study, the aim was to study the stable development of new ventures at the present stage from the two aspects, namely, managers (especially new venture entrepreneurs) and enterprise employees. QS and SPSS 25.0 software are used for data statistical analysis. The main conclusions are as follows: first, the QS results of college athletes show that there is a significantly positive correlation between the career guidance curriculum and student employability. Second, the career planning, vocational training, and internship assistance of the career guidance curriculum are positively correlated with the employment success rate, career preference, and job satisfaction of student employability. Analysis of the mechanism of psychological capital between the career guidance curriculum and student employability can provide theoretical references and suggestions for the successful employment of students and the educational model and management system of college athletes in colleges and universities. Third, the QS results of new venture entrepreneurs show that appropriate psychological stress can have a significant correlation with the new venture performance. Specifically, entrepreneurship involved, competition intensity, resource requirements, and knowledge reserve stress have positive effects on the survival performance, growth performance, and development performance of new ventures. The experimental results have fully considered the important factors related to the development of new ventures, and the results are comprehensive and have some practical reference value. There are also some limitations as follows: the scope of the QS is small, and there is no targeted long-term follow-up discussion. In the follow-up research, the scope of the research will be further expanded and the research time will be increased, which is of a more practical reference value.

## Data Availability Statement

The original contributions presented in the study are included in the article/supplementary material, further inquiries can be directed to the corresponding author/s.

## Ethics Statement

The studies involving human participants were reviewed and approved by University of Malaya Ethics Committee. The patients/participants provided their written informed consent to participate in this study. Written informed consent was obtained from the individual(s) for the publication of any potentially identifiable images or data included in this article.

## Author Contributions

All authors listed have made a substantial, direct and intellectual contribution to the work, and approved it for publication.

## Conflict of Interest

The authors declare that the research was conducted in the absence of any commercial or financial relationships that could be construed as a potential conflict of interest.

## Publisher's Note

All claims expressed in this article are solely those of the authors and do not necessarily represent those of their affiliated organizations, or those of the publisher, the editors and the reviewers. Any product that may be evaluated in this article, or claim that may be made by its manufacturer, is not guaranteed or endorsed by the publisher.
